# Overexpression of Salusin-*α* Inhibits Vascular Intimal Hyperplasia in an Atherosclerotic Rabbit Model

**DOI:** 10.1155/2018/8973986

**Published:** 2018-07-12

**Authors:** Kun Qian, Li Feng, Yujie Sun, Bowen Xiong, Yi Ding, Panting Han, Hailun Chen, Xiao Chen, Ling Du, Yuxue Wang

**Affiliations:** ^1^Department of Laboratory Medicine, Hubei University of Chinese Medicine, Wuhan, Hubei, China; ^2^Endoscopy Center, Minhang Branch of Zhongshan Hospital, Fudan University, Shanghai, China

## Abstract

Inhibiting vascular endothelial foam is the focus of clinical attention. Using SonoVue (an ultrasound contrast agent), the salusin-*α* gene was transfected into the arterial intima of an atherosclerotic rabbit model induced by a high-fat diet in this study. Subsequently the model of blood lipid indexes, the pathological structure of the intima, and changes in molecules regulating atherosclerosis were investigated. The high-density lipoprotein C and apolipoprotein A values in the salusin-*α* gene overexpression (P) group were higher than those in the salusin-*α* gene interference (RP) group (*P* < 0.05), whereas the total cholesterol, low-density lipoprotein C, and apolipoprotein B values were reversed. Rabbits in the P group showed significantly thinner vascular intimal thickness than that of other experimental groups (*P* < 0.05). The expression of positive regulators of atherosclerosis (*ABCA1*,* ABCG1*) was higher in the P group than that in the RP group (*P* < 0.05), and the opposite effect was observed for negative regulators (*ACAT1*,* CD36*). Thus, our results showed that the overexpression of salusin-*α* gene inhibited the proliferation of the vascular intima, thereby throwing some light on understanding the mechanism how salusin-*α* gene expression interfered with the foaming of vascular intimal cells.

## 1. Introduction

Atherosclerosis is one of the most common causes of vascular disease [1, 2] and is characterized by thickening of the artery walls, loss of elasticity, and narrowing of the lumen. Surgical treatment of stenosis or occlusion of blood vessels to restore arterial blood supply is a common approach to the management of this disease [3, 4]; however, postoperation intimal hyperplasia and vascular stenosis remain unresolved problems in the treatment of vascular disorders [3]. Clinically, catheter-based interventions, including balloon angioplasty and stenting, initially restored blood flow in obstructed arteries in more than 95% of patients [5, 6]. Within 6 months, however, vasospasm, thrombosis, and intimal hyperplasia significantly induce restenosis of the arterial blood vessels in 15–40% of treated patients [3, 7]. The pathogenesis of intimal hyperplasia after vascular injury is believed to be a serious problem for long-term and adequate blood supply by arterial blood vessels [8, 9]. Thus, researchers are currently focusing on prevention of the restenosis of the arterial vascular wall.

Salusin-*α* is derived from the torsin family 2 member A (*TOR2A*) gene, which is transcribed into preprosalusins and subjected to alternative splicing. Salusin-*α* is a multifunctional bioactive peptide with 28 amino acid residues that functions to prevent the restenosis of arteries [10, 11]. In addition, this protein is also involved in the prevention of macrophage foam cell formation by downregulating acetyl-coenzyme A acetyltransferase 1 (ACAT1), thereby affecting atherosclerosis [10, 12, 13]. Some reports have shown the negative correlation between salusin-*α* levels in serum and the degree of vascular stenosis, suggesting that salusin-*α* may have applications as a diagnostic reference in atherosclerotic disease [14, 15]. However, the mechanisms through which salusin-*α* regulates atherosclerosis in vivo are unclear.

Accordingly, in this study, we investigated the effects of salusin-*α* on vascular atherosclerosis through continuous transfection of salusin-*α* into the vessel wall in an atherosclerotic rabbit model.

## 2. Materials and Methods

### 2.1. Construction of pcDNA3.1-Salusin-*α* and psiHIV-U6-Salusin-*α*

According to a previous protocol [16], the 84-bp human salusin-*α* gene (synthesized by Sangon Biotech Co., Ltd., Shanghai, China) was inserted into compatible enzyme restriction sites (*Xho*I and* BamH*I) of pEGFP-N1 (Invitrogen Life Technologies, Carlsbad, CA, USA) to generate pEGFP-salusin-*α*. The forward primer was TGTGGGATCCATGATTTACACAATGAAG, and the reverse primer was TATACTCGAGCGACATAGGCATGAAATGCTATC. The polymerase chain reaction (PCR) product of the salusin-*α* gene was digested with restriction enzymes* BamH*I and* Xho*I, and the desired fragment was cloned into the pcDNA3.1 expression plasmid (Invitrogen Life Technologies) to generate pcDNA3.1-salusin-*α* (pc-salusin-*α*), which the salusin-*α* gene could be overexpressed by the pc-salusin-*α* plasmid transfected cells. Similar to our original study, the RNA interference psiHIV-U6-salusin-*α* (si-salusin-*α*) recombinant was constructed as described using a Lenti-Pac lentiviral packaging kit (GeneCopoeia, MD, USA). The salusin-*α* of si-salusin-*α* indicated that the interference target sequence of salusin-*α* gene was tccggcactgcgtgctcaa. The si-salusin-*α* transfected cells might inhibit the expression of the salusin-*α* gene. Constructed plasmids were confirmed by DNA sequencing. High-purity plasmids were prepared on a large scale in the experiments described below.

### 2.2. Animal Model and Grouping

All healthy New Zealand white rabbits of both sexes (~2.5 kg) were obtained from the Center of Medical Experimental Animals (Hubei Province, China). Animals were maintained in our laboratory under standard conditions. All rabbits were treated under protocols approved by the Institute of Animal Care and Use Committee of Hubei University of Chinese Medicine (approval documents number 2015025, time: 2015-6-3). We started to use a high-fat diet-induced atherosclerosis vascular model [17]. All rabbits were fed a high-fat diet every day, except for rabbits in the ordinary diet (OD) group. The high-fat diet formula increased 15% egg yolk powder, 0.5% cholesterol, and 5% lard on the basis of the OD, and the OD formula is constituted by the main food production of corn based food particles (including corn 47%, wheat bran 20%, soybean meal 25%, fish meal 5%, shell powder 2%, salt 1%, and compound vitamin 0.05%). All rabbits to the dietary supplements were fed 125 g of a commercial feed daily. Water purified by reverse osmosis was supplied through an automated watering system to the cages. The room temperature was set at 68°F(20°C), humidity to a range of 45% to 50%, and a 12:12-h light:dark cycle. As described in the Guide and the Animal Welfare Act Regulations, the rabbits were housed in caging constructed of stainless steel and that met the standards for interior floor square surface area. The collection pans underneath the cages were lined with the shepherd specialty papers which were changed twice weekly. The entire cage was changed and sanitized biweekly. The racks were situated to allow visual, auditory, and olfactory stimulation for all rabbits in the room. After 6 weeks, the carotid artery diameter and blood flow velocity of rabbits were detected by ultrasound for the various groups. Then, all rabbits were put to death by the anaesthesia method for obtaining the carotid arteries. The method of anaesthesia was to increase the dose of pentobarbital gradually by ear intravenous injection until the rabbit stops the heartbeat. In order to alleviate the pain in rabbits, rabbits were prevented from observing the execution process before sacrifice.

A total of 42 rabbits were used for analysis of the effects of salusin-*α* on the vascular intimal hyperplasia of arteries and were divided into seven groups: the salusin-*α* gene overexpression of pc-salusin-*α* plasmid (P) group (n = 6), the salusin-*α* gene RNA interference of si-salusin-*α* plasmid (RP) group (n = 6), the pcDNA3.1 empty plasmid (EP) without inserting the salusin-*α* gene group (n = 6), the psiHIV-U6 empty RNA plasmid (ERP) without inserting the interference target sequence of salusin-*α* gene group (n = 6), the high-fat diet (HFD) group (n = 6), the SonoVue (S) group (n = 6), and the OD group (n = 6). In order to overexpress or inhibit the salusin-*α* gene in vascular endothelial cells, rabbits in the P and RP groups were transfected with pc-salusin-*α* and si-salusin-*α* plasmid, respectively. In contrast, rabbits in the EP and ERP groups were severally transfected with pcDNA3.1 and psiHIV-U6 plasmids, which were not inserted by the salusin-*α* gene and interference target sequence, respectively. Rabbits in the HFD group did not receive any treatment except high-fat diet feeding. Rabbits in the S group were injected with phosphate-buffered saline (pH 7.4) and SonoVue (Bracco Diagnostics, Inc., Stony Brook, NY, USA) and then subjected to ultrasound. Finally, rabbits in the OD group were fed an ordinary diet throughout the entire experimental program.

During the course of the experiment, plasmid transfection was carried out once a week. The method of plasmid transfection was the same as that in our original study [16]. In brief, we firstly anaesthetized all rabbits into the coma state using the pentobarbital sodium (25 mg/kg; Invitrogen) by ear intravenous injection. A mixture of 100 mg of plasmids (1 mg/mL) and 200 mL of SonoVue (45 mg/mL) was incubated for 20 min at room temperature and was injected into the ear vein through a 22-gauge intravenous cannula, followed by a flush of 2 mL of 0.9% normal saline solution. After injection, the right common carotid artery was quickly sonoporated for 3min using ultrasound until the SonoVue microbubbles had been largely disrupted from the circulatory system.

### 2.3. Baseline and Contrast-Enhanced Ultrasonography Examination Settings [16]

A clinical ultrasound scanner (Siemens Acuson Sequoia 512, Siemens, Germany) and a convex probe with a frequency range of 10–14 MHz (15L8W-S, Siemens) were used in the present study. For baseline ultrasound examination, the conventional carotid artery examination mode was used, and the mechanical index (MI) was 1.5 as read on the screen. The carotid artery was scanned thoroughly with baseline ultrasound for five minutes. The imaging settings of the ultrasound scanners were optimized to get the best depiction of the carotid artery (Table 1). For low MI real-time contrast-enhanced ultrasonography (CEUS) examination, the contrast pulse-sequencing imaging mode was used and MI was 0.18 as read on the screen. SonoVue was used as a contrast agent. After the initiation of contrast-specific imaging mode, the imaging settings, including the depth and focus, were readjusted for best visualization of the carotid artery (Table 1). A volume of 0.2–0.3 mL (0.1 mL/kg) of a contrast agent was injected into the ear vein in 10 s, followed by a flush of 2 mL of 0.9% normal saline solution. No animal received an additional administration of the contrast agent in this study. The timer was activated simultaneously at the beginning of ultrasound contrast agent (UCA) administration. The carotid artery was first observed continuously for five minutes after UCA administration as a vascular phase, and then from the 10th to 15th min as a Kupffer phase under contrast-specific imaging, and high MI (1.5) imaging mode for three minutes to break the microbubbles in the end. The digital cine clips of both baseline grey-scale ultrasound and the whole process of CEUS images were stored in the hard disk incorporated in the scanner.

### 2.4. Blood Lipid Examination

The blood of the rabbits anaesthetized as above was obtained from ear vein before sacrifice, and blood lipids were detected using automatic biochemical analyzer (UniCel DxC 800; Beckman Coulter, Miami, FL, USA). Blood lipid testing included triglycerides (TGs), total cholesterol (TC), high-density lipoprotein cholesterol (HDL-C), low-density lipoprotein cholesterol (LDL-C), apolipoprotein A (ApoAI), apolipoprotein B (ApoB), and lipoprotein alpha [Lp(*α*)]. All test items of specimens were detected three times according to the laboratory standard.

### 2.5. Histopathological and Arterial Tension Analysis

Histopathological examination of tissue sections was performed to investigate vascular intimal hyperplasia. Similar to routine pathological examination [3, 16], paraffin-embedded blocks of the carotid artery tissue were taken from each rabbit group, and paraffin-embedded tissues were continuously sliced into 4-*μ*m thick sections, which were subsequently stained with hematoxylin and eosin (HE) and examined by a trained pathologist. Magnified images were captured using an Axiophot microscope (Zeiss, Austin, TX, USA) and a digital camera (Leaf Systems Lumina, Southborough, MA, USA). Images were processed with software from Optima Imaging Analysis Systems (Version 6.5, Media Cybernetics, Silver Springs, MD, USA).

Examination of arterial tension (Dual Wire Myograph System-410A; DMT-USA Inc., Atlanta, GA, USA) was used to estimate artery function. Detection of arterial tension was carried out as previously reported [3]. Acetylcholine- (ACh-) stimulated endothelial cells produce nitric oxide, which causes smooth muscle relaxation. Thus, by testing arterial tension, arterial endothelial function could be indirectly measured. Accordingly, in this study, changes in the tension of the arteries in the experimental groups and the control groups by different concentrations of ACh were used to determine arterial function in each group.

### 2.6. Western Blot Analysis

For protein expression analysis, western blotting was performed as previously described [3, 16]. The carotid arteries used for western blotting were homogenized in 1 mL lysis buffer containing 50 mM NaCl, 10 mM Tris, 1 mM ethylenediaminetetraacetic acid, 1 mM phenylmethylsulfonyl fluoride, 0.5 mM Na_3_VO_4_·12H_2_O, 50 mM NaF, and 1 mM benzamidine. The samples were centrifuged at 12500 ×* g* for 15 min at 4°C. The protein concentration was determined using BCA Protein Assay Reagents (Pierce Com., Rockford, IL, USA). Equal amounts of lysate protein (20 *μ*g) for each sample were loaded on 7.5% sodium dodecyl sulfate-polyacrylamide gels for electrophoresis and then blotted onto polyvinylidene difluoride membranes (Amersham Pharmacia Biotech, Piscataway, NJ, USA). Nonspecific binding to the membranes was blocked with 3% bovine serum albumin in Tris-buffered saline for 1 h at room temperature. They were then incubated with different primary antibodies overnight at 4°C, as follows: anti-salusin-*α* rabbit polyclonal antibodies (Yansheng, Shanghai, China; cat. no. YS-KT2539), anti-enhanced green fluorescent protein (EGFP) rabbit polyclonal antibodies (OpenBiosystems, Ohio, USA; cat. no. CAB4211), anti-ABCA1 rabbit polyclonal antibodies (Novus Biologicals, Littleton, CO, USA; cat. no. NB400-105), anti-ABCG1 rabbit polyclonal antibodies (Novus Biologicals; cat. no. NB400-132), anti-ACAT1 rabbit polyclonal antibodies (Novus Biologicals; cat. no. AF2222a), anti-CD36 rabbit polyclonal antibodies (Novus Biologicals; cat. no. NB400-144), and anti-glyceraldehyde-3-phosphate dehydrogenase (GAPDH; Santa Cruz, CA, USA; cat. no. sc-25778). Horseradish peroxidase-conjugated secondary goat anti-rabbit IgG2a antibodies (Santa Cruz; cat. no. sc-2061) were used as secondary antibodies. Western blots were visualized using enhanced chemiluminescence detection reagents (Sigma, St. Louis, MO, USA) according to the manufacturer's instructions. Quantification of protein bands was performed via scanning with Bio-Rad GelDoc XR and Chemi Doc XRS systems (Bio-Rad, Hercules, CA, USA), and the bands were analyzed using Quantity One 1-D Analysis Software Version 4.6.3 (Bio-Rad).

## 3. Statistical Analysis

All data are presented as means ± standard deviations. For statistical analysis, SPSS 19.0 software (SPSS, Chicago, IL, USA) was used, and the mean results of different groups were analyzed by ANOVA to determine statistical significance. Differences with* P* values of less than 0.05 were considered significant. All experiments were carried out three times.

## 4. Results

### 4.1. Analysis of Model Establishment and Plasmid Transfection

**Figure 1:**
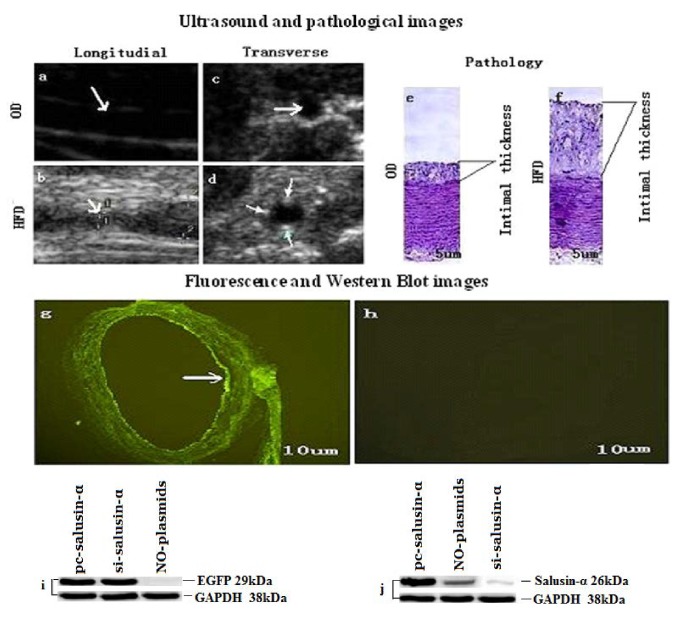
Analysis of model establishment and plasmid transfection. Representative ultrasound images (a–d) are longitudinal and transverse sections of the arteries in the HFD and OD groups (the white arrows indicate the lumen of the arteries). Representative pathological images ((e) and (f)) show the average thickness of the arterial intima in the HFD and OD groups. Representative fluorescence and western blot images (g-j) of arteries transfected by plasmids. The white arrows (g) indicate the intima transfected by the two plasmids (pc-salusin-*α* and si-salusin-*α*) with the* EGFP* reporter gene in the transfection groups, and the image in (h) indicates the arterial wall transfected by no plasmids. The western blot image (i) shows the EGFP gene expression in three different groups, which were Salusin-alpha gene overexpression (pc-salusin-*α*), salusin-alpha gene interference (si-salusin-*α*), and no plasmids transfection (NO-plasmid) group. Similarly, the salusin-alpha gene expression in the three groups was displayed by (j). The data are representative of three independent experiments (n = 3).

In order to verify successful establishment of the model, we detected the carotid arterial intima by ultrasound and pathological methods in the OD and HFD groups. Compared with the common carotid arteries of the two groups, we found that there was no plaque in the longitudinal and transverse sections of the arteries in rabbits in the OD group (Figures 1(a) and 1(c)). However, in the HFD group, plaques and lumen stenosis were detected in the carotid artery (Figures 1(b) and 1(d)). Pathological examination revealed thickening of the carotid arterial intima in the HFD group compared with that of the OD group (Figures 1(e) and 1(f)). These results showed that the atherosclerosis vascular model was successful.

Before the start of the study, we used fast frozen sections to examine whether the constructed plasmids (pc-salusin-*α* and si-salusin-*α*) could be transferred to the arterial wall. Based on previous methods [3, 16], the two constructed plasmids with EGFP (a green fluorescent protein gene) were transfected into the arterial wall, and the fluorescence of the arterial wall was tested by fluorescence microscopy. Strong fluorescence was detected in the intima of the arterial wall in transfected samples (Figure 1(g)), whereas no specific fluorescence was observed in the carotid artery of untransfected plasmid samples (Figure 1(h)). Similarly, western blotting showed that EGFP was detected in transfected plasmids (pc-salusin-*α* and si-salusin-*α*) samples but not in untransfected plasmid samples (Figure 1(i)). As shown in Figure 1(j), the arterial intima was then transfected with the pc-salusin-*α* plasmids harboring the salusin-*α* gene; salusin-*α* expression was lower in untransfected plasmids tissues than in pc-salusin-*α* plasmids transfected tissues, but higher si-salusin-*α* plasmid transfection tissues. Thus, these results demonstrated that the plasmids harboring the target gene had been successfully transfected into the arterial intima.

### 4.2. Blood Lipid Detection in Different Groups

To evaluate the effects of salusin-*α* on blood lipid metabolism, we tested seven blood lipid parameters, i.e., TGs, TC, HDL-C, LDL-C, ApoAI, ApoB, and Lp(*α*), in different groups (Table 2). There were no differences in Lp(*α*) levels among groups (*p *> 0.05). The TG value in the OD group was significantly lower than those in the other groups (*p *< 0.01), but there were no other significant differences among groups (*p *> 0.05). TC and LDL-C levels were the highest in the RP group and the lowest in the P group; moreover, TC and LDL-C levels were lower in the OD group than in the EP, ERP, HFD, and S groups (*p *< 0.05), which were considered control groups. In fact, there were no differences in all seven blood lipid items among the four control groups (*p *> 0.05). The results of HDL-C and ApoAI levels showed trends opposite those for TC and LDL-C in the seven groups. Additionally, ApoB levels were higher in the OD group than in the P group (*p *< 0.05) and were lower in the OD group than in the other five groups (*p *< 0.01). HDL-C and ApoAI levels were higher in the P group than in the other groups (*p *< 0.01), whereas ApoB levels were significantly lower in the P group than in the other groups. The results in the RP group were opposite those in the P group. From these data, we concluded that the increase in salusin-*α* expression altered lipid metabolism in vivo.

### 4.3. Effects of Salusin-*α* Gene Transfer into Rabbits on Intima Media Thickness

**Figure 2:**
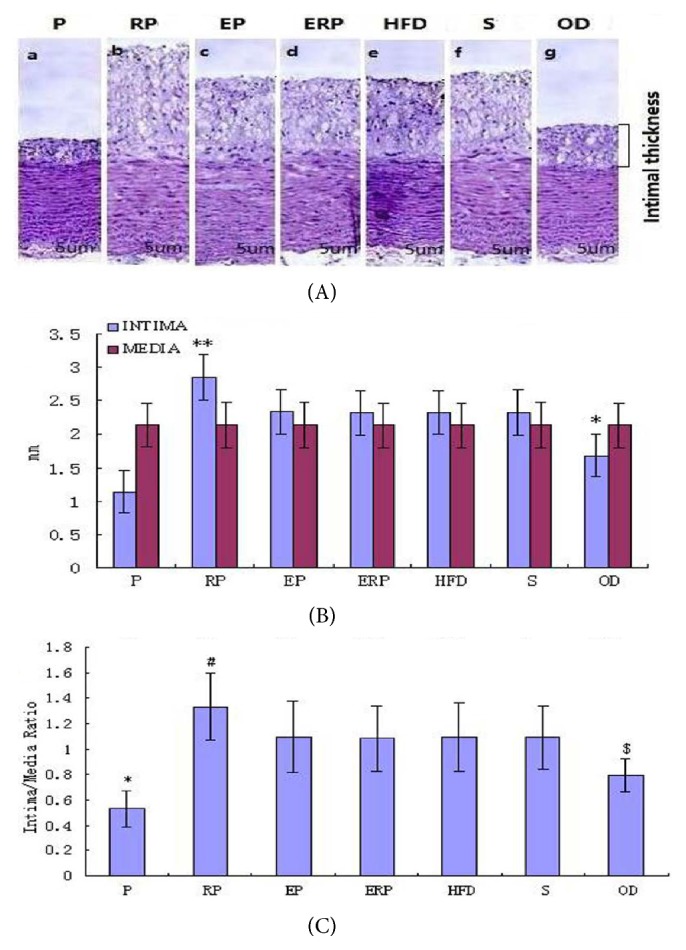
Effects of the salusin-*α* gene on model intima media thickness. (A) Representative average arterial intima thickness images (a-g) in these seven groups. (B) The blue column indicates the mean arterial intima thickness in the seven groups, and the red column indicates the media. (C) The mean intima/media ratios were calculated as the mean arterial intima thickness divided by the media. The data are representative of three independent experiments (n = 3)(*∗∗p* < 0.01, *∗p* < 0.05).

In accordance with previous methods [3], we examined the inhibitory effects of pcDNA3.1-salusin-*α* transfection on intimal hyperplasia after administration of a high-fat diet in a rabbit carotid artery model. As shown in Figure 2, the salusin-*α* gene overexpression significantly suppressed intimal hyperplasia in the carotid artery models.

At 6 weeks after consumption of a high-fat diet, we measured the intima/media areas ratio using histopathology. Because the salusin-*α* gene expression was suppression in RP group, the intima of the arteries was the thickest, and that from the P group was opposite. The intimal thickness was lower in the OD group than in the four control groups, which did not differ (Figure 2(A)). The mean intima thicknesses of the arteries in the P, RP, EP, ERP, HFD, S, and OD groups were 1.14 ± 0.32, 2.85 ± 0.34, 2.34 ± 0.33, 2.32 ± 0.33, 2.33 ± 0.33, 2.33 ± 0.34, and 1.68 ± 0.32 mm, respectively. There were significant differences between the P group and other groups (*p *< 0.05; Figure 2(B)). The mean intima/media ratio of the salusin-*α*-transfected carotid arteries was smaller 80% (*p *< 0.01) than that of RP group and the other groups. There were no differences in the mean intima/media ratio among the four control groups, and that of the controls was lower than that in the RP group and higher than that in the OD group (Figure 2(C),* p *< 0.05). The results indicated that overexpression of the salusin-*α* gene reduced the thickening of the intima.

### 4.4. Effects of Salusin-*α* on Arterial Diameter, Blood Flow Velocity, and Arterial Tension in Rabbits

**Figure 3:**
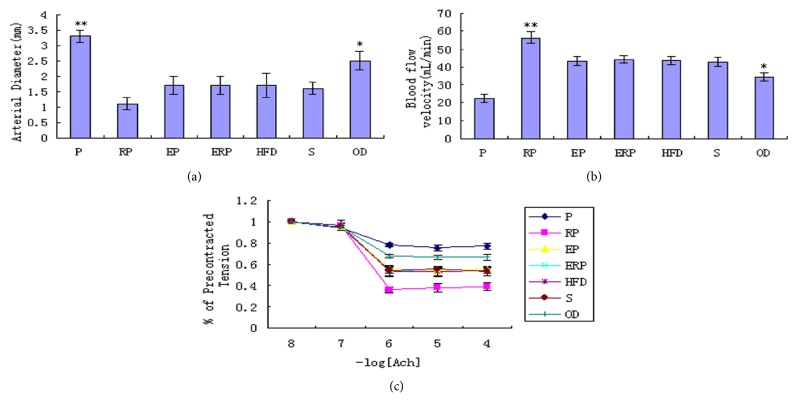
Effects of salusin-*α* on arterial diameter, blood flow velocity, and arterial tension. (a) The average arterial diameter was detected in the seven groups. (b) The mean blood flow velocity was detected in the seven groups. (c) The mean % of precontracted tension was calculated in the seven groups. The data are representative of three independent experiments (n = 3) (*∗∗p* < 0.01, *∗p* < 0.05).

Next, we examined arterial diameter and blood flow velocity by ultrasound analysis. Arterial diameter was the largest in the P group and the smallest in the RP group. However, the arterial diameters of the four control groups did not differ (*p *> 0.05) and were smaller in the control groups than in the OD group (Figure 3(a),* p *< 0.05). In contrast, the arterial blood flow velocity showed the opposite trends (Figure 3(b)).

The arterial tension changed with ACh dose in the seven groups (Figure 3(c)). The arterial tension in the RP group was lower than that in the other groups (*p *< 0.05), and that in the P group was the highest. As shown in Figure 3(a), the arterial tension in the OD group was slightly lower than that in the P group.

Taken together, these data suggested that the salusin-*α* gene increased the diameter and tension of the arterial vessels and reduced the flow of blood.

### 4.5. Effects of Salusin-*α* Overexpression on the Levels of ABCA1, ABCG1, ACAT1, and CD36

**Figure 4:**
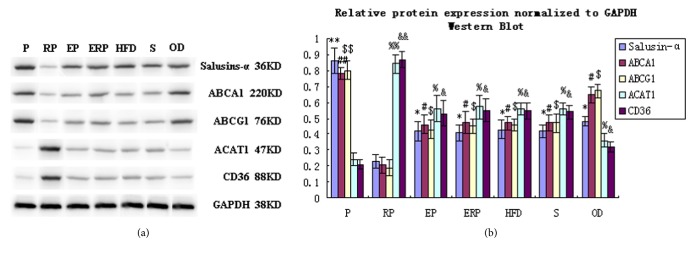
Effects of salusin-*α* overexpression on the levels of ABCA1, ABCG1, ACAT1, and CD36. (a) Representative western blotting results in the seven groups. (b) The columns indicated the mean western blot band densities in the seven groups. The data are representative of three independent experiments (n = 3). The distinct symbols indicate the comparison mean of different protein expression levels between different groups. *∗∗p* < 0.01, *∗p* < 0.05; ^##^*p* < 0.01, ^#^*p* < 0.05; ^$$^*p* < 0.01, ^$^*p* < 0.05; ^%%^*p* < 0.01, ^%^*p* < 0.05; ^&&^*p* < 0.01, and ^&^*p* < 0.05.

ABCA1, ABCG1, ACAT1, and CD36 are positive or negative regulators of atherosclerosis. In order to explore the relationship between these regulators and salusin-*α* expression, we used western blotting to analyze the effects of salusin-*α* on the expression of regulators in rabbit arterial vessels. The levels of ABCA1 and ABCG1 were significantly higher in the P group than in the other groups (*p *< 0.05) and significantly lower in the RP group than in the other groups. Moreover, ACAT1 and CD36 expression levels showed opposite trends in the P and RP groups. For the control groups, there were no differences in the expression levels of the four genes (Figures 4(a) and 4(b);* p *> 0.05). These results revealed that overexpression of salusin-*α* inhibited the expression of ACAT1 and CD36 but increased the levels of ABCA1 and ABCG1.

## 5. Discussion

This study revealed that overexpression of salusin-*α* inhibited intimal hyperplasia in atherosclerotic rabbits induced by a high-fat diet. Moreover, overexpression of salusin-*α* regulated lipid metabolism, enhanced the expression of positive regulatory molecules in atherosclerosis, and inhibited the expression of negative regulatory molecules, thereby suppressing the proliferation of the vascular intima.

The high-fat diet-induced atherosclerosis model has been successfully established in previous studies [17, 18]. In this study, we aimed to study an atherosclerosis rabbit model; to this end, we used induction of atherosclerosis by consumption of a high-fat diet in ApoE-deficient mice [19, 20]. Although the high-fat diet induced atherosclerosis quickly in ApoE-deficient mice, deletion of the* ApoE* gene may alter the results of the study [21, 22]. Notably, in our study, the arterial intima became thicker in the HFD group, whereas that in the OD group did not change. After the rabbit model was established, the intima of the HFD group was thicker than that of the OD group. This confirmed the establishment of atherosclerosis induced by the high-fat diet [17, 18].

Ultrasound contrast agents have been used to deliver drugs or plasmids [23–26]. This transfer process usually involves a drug or plasmid coated by the ultrasound contrast agent. However, we found that SonoVue and the plasmid showed strong adhesion; therefore, we used SonoVue to transfer the plasmid into the rabbit common carotid arterial intima [3, 16]. This method was shown to be successful for transfecting tissues with the target plasmid expressing salusin-*α*, enabling us to further study the role of salusin-*α* in atherosclerosis. Importantly, we found that overexpression of the salusin-*α* gene decreased the intima thickness in rabbits. Intimal hyperplasia in atherosclerotic rabbit models is usually caused by excessive accumulation of intracellular lipids by intimal cells [27–29], and the salusin-*α* gene has been reported to reduce hypertension and inhibited macrophage foam [14, 15]. Thus, the finding that overexpression of salusin-*α* decreased intimal thickness could be related to inhibition of the proliferation of vascular endothelial cells.

Routine examination of blood lipid parameters is mainly used to determine lipid metabolism in the human body [30, 31]. Long-term consumption of a high-fat diet could alter the lipid metabolism parameters in rabbits [32–34]. Our findings showed that salusin-*α* gene expression regulated lipid metabolism and prevented the formation of atherosclerosis. We also examined artery diameter, blood flow velocity, and arterial tension, which are the main indicators reflecting the functional status of blood vessels [16, 17, 35]. Our results showed that salusin-*α* overexpression increased arterial diameter and decreased blood flow velocity, consistent with a previous study showing that blood vessel diameter and blood flow velocity are inversely proportional [36]. Furthermore, the mean arterial tension was significantly higher following overexpression of salusin-*α*. Taken together, these findings demonstrated that overexpression of salusin-*α* improved the properties of arteries in rabbits.

The salusin-*α* gene inhibits atherosclerosis [10, 15], and detection of the salusin-*α* gene may be used as a reference index for the diagnosis and treatment of cardiovascular disease [15]. Therefore, we investigated the mechanisms through which salusin-*α* modulates atherosclerosis by examining the expression of positive and negative regulators of atherosclerosis in the vascular intima [37–39]. Our results showed that overexpression of salusin-*α* increased the expression of positive regulators of atherosclerosis (ABCA1 and ABCG1) and inhibited negative regulators of atherosclerosis (ACAT1 and CD36). This result revealed that overexpression of salusin-*α* altered the progression of atherosclerosis. However, further studies are needed to determine the specific mechanisms through which salusin-*α* regulates atherosclerosis.

## 6. Conclusions

In this study, we found that salusin-*α* modulated positive and negative regulators of atherosclerosis and prevented intimal hyperplasia. These data provide a reference for further studies of the mechanisms through which salusin-*α* affects vascular intimal foam and for the potential clinical applications of this target. However, as there are many causes of atherosclerosis, the intervention of atherosclerosis needs to be further studied by multiple factors.

## Figures and Tables

**Table 1:** Setting of ultrasound scanner used in the study.

Parameter	Baseline examination	CEUS examination
Machine type	Siemens Acuson Sequoia 512 15L8W-S probe	
Imaging mode	2-D imaging	Contrast pulse sequencing imaging
MI (as read on screen)	1.5	0.18; 1.5
Frequency (MHz)	10.0–14.0	10.0–14.0
Depth (cm)	2–2.5	2–2.5
Focus position (cm)	0.5–1	0.5–1
Focus number	1	1

Parameter setting of ultrasound scanner in the study CEUS, contrast-enhanced ultrasonography; MI, mechanical index.

**Table 2:** Blood lipid biochemical parameters in different groups.

Group	P	RP	EP	ERP	HFD	S	OD
TG (mmol/L)	3.45 ± 0.72	3.35 ± 0.74	3.56 ± 0.52	3.63 ± 0.64	3.43 ± 0.82	3.12 ± 0.56	1.12 ± 0.64*∗∗*

TC (mmol/L)	0.83 ± 0.91*∗∗*	3.68 ± 0.53	2.54 ± 0.42	2.42 ± 0.37	2.63 ± 0.35	2.61 ± 0.33	1.63 ± 0.86*∗*

HDL-C (mmol/L)	1.42 ± 0.12*∗∗*	0.21 ± 0.08	0.35 ± 0.06	0.34 ± 0.07	0.37 ± 0.09	0.36 ± 0.10	0.52 ± 0.14*∗*

LDL-C (mmol/L)	0.37 ± 0.08*∗∗*	2.64 ± 0.76	1.54 ± 0.55	1.64 ± 0.72	1.44 ± 0.52	1.54 ± 0.62	0.78 ± 0.57*∗*

ApoAI (g/L)	0.43 ± 0.06*∗∗*	0.10 ± 0.02	0.16 ± 0.02	0.17 ± 0.02	0.16 ± 0.01	0.15 ± 0.02	0.18 ± 0.04

ApoB (g/L)	0.03 ± 0.01*∗∗*	0.13 ± 0.03	0.11 ± 0.03	0.12 ± 0.01	0.14 ± 0.02	0.13 ± 0.02	0.05 ± 0.01*∗*

Lp (*α*) (mg/L)	187 ± 30.7	178 ± 27.6	189 ± 30.3	187 ± 28.5	188 ± 27.9	191 ± 30.2	193 ± 28.5

Variables are presented as means ± standard deviations. P, pc-salusin-*α* plasmid group; RP, RNA plasmid si-salusin-*α* group; EP, empty plasmid group; ERP, empty RNA plasmid group; HFD, high-fat diet group; S, SonoVue group; OD, ordinary diet group; TG, triglyceride; TC, total cholesterol; HDL-C, high-density lipoprotein cholesterol; LDL-C, low-density lipoprotein cholesterol; ApoAI, apolipoprotein A; ApoB, apolipoprotein B; and Lp(*α*), lipoprotein alpha. *∗∗p* < 0.01, *∗p* < 0.05.

## Data Availability

The data used to support the findings of this study are available from the corresponding author upon request.
